# Unexpected Acute-Onset Stump Appendicitis in a Teenager: A Rare Postappendectomy Complication

**DOI:** 10.1155/cris/3546440

**Published:** 2025-08-22

**Authors:** Ahmad Fasfoos, Maaweya Jabareen, Wasef Alhroub, Ammar Hassouneh, Dunia Salhab, Aleen Aldabbas, Razan Sobeih, Isra Hamoudah, Islam Ishnawer, Qusai Sobeih

**Affiliations:** ^1^Department of Surgery, Hebron University, Hebron, West Bank, Palestinian Territory, State of Palestine; ^2^Department of Surgery, Alexandria University, Alexandria, Alexandria Governorate, Egypt

**Keywords:** abdominal pain, incomplete appendectomy, recurrent appendicitis, stump appendicitis, surgical complications

## Abstract

Stump appendicitis is a rare but serious complication following an appendectomy, resulting from incomplete removal of the appendix. It often mimics acute appendicitis with nonspecific symptoms, such as abdominal pain, nausea, vomiting, and fever, making diagnosis challenging. Here, we report the case of a 17-year-old male who presented with diffuse abdominal pain, fever, and nausea just 11 days after undergoing an open appendectomy. Physical examination revealed tenderness at the surgical site, and imaging showed inflammatory changes in the right lower quadrant. Exploratory surgery confirmed stump appendicitis due to retained appendiceal tissue, which was resected. The patient recovered uneventfully.

## 1. Introduction

The appendectomy is one of the most common surgical procedures performed [1]. Stump appendicitis is an infrequent but serious complication following appendectomy. The incidence is approximately 1 in 50,000 [2]. The condition is a result of obstructed and inflamed retained appendiceal tissue after incomplete resection of the appendix. Certainly, incomplete resection is more common in laparoscopic procedures due to the great tendency of leaving appendiceal remnants [3, 4].

Diagnosis of stump appendicitis is often challenging, as most people are presenting symptoms similar to acute appendicitis: abdominal pain, nausea, vomiting, and leukocytosis. This delays the appropriate diagnosis of the condition [4]. The problem may appear several months or years later after the surgery is performed, and in most of the cases, perforation, among other complications, occurs, adding to morbidity [3].

Early detection of stump appendicitis is very critical in preventing severe complications, which include peritonitis or the development of abscesses and sepsis [2]. Common diagnostic tests applied in the diagnosis of the disease involve imaging; among them, abdominal CT is mostly used. The mainstay of treatment involves a complete appendectomy, a surgical procedure intended to remove the residual appendiceal tissue [5].

This case presents a 17-year-old with diffuse abdominal pain after 11 days of open appendectomy, with fever and nausea. Exploratory surgery identified and removed retained appendiceal tissue, confirming the stump appendicitis.

## 2. Case Presentation

A 17-year-old male presented to the emergency department with diffuse abdominal pain lasting for 2 days that was initially intermittent and localized, then gradually progressed to constant and generalized. In addition, the patient complained of nausea and vomiting without any additional symptoms. His surgical history included open appendectomy for acute appendicitis 11 days ago. On clinical examination, he appeared tired and dehydrated. He exhibited a fever of 40°C but was otherwise vitally stable. Local examination of the abdomen revealed a soft and lax abdominal wall with tenderness at the surgical site. Laboratory tests on admission revealed: hemoglobin was 10.1 g/dL; total white cell count was 9500 mm^3^ (89% neutrophils).

**Figure 1:**
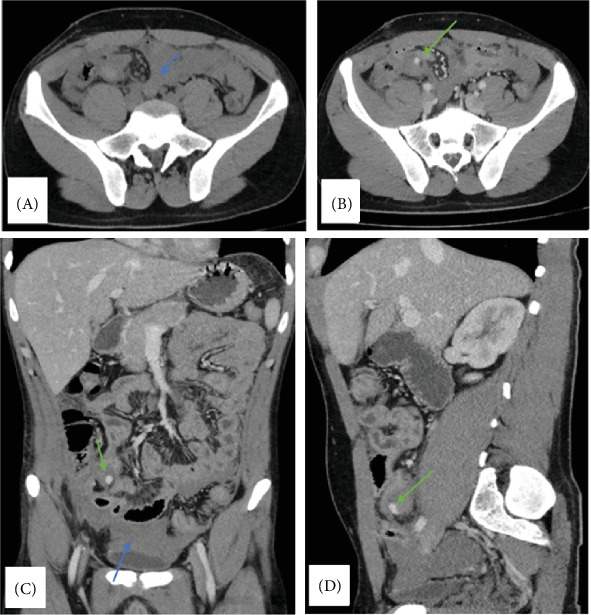
CT abdomen without and with IV contrast-portal phase; in axial (A, B), coronal (C), and sagittal (D) planes, moderate amount of free fluid with high density suggestive of hemoperitoneum with clots formation was noted predominantly at right iliac fossa (blue arrow), in addition, focal hyper dense area was noted on post contrast images at site of removed appendiceal stump raise the possibility of peritoneal bleeding related to appendiceal stump (green arrow).

Abdominal ultrasound findings diffuse an increase in small bowel wall thickness, mainly in the RLQ, with a significant increase in the echogenicity of the surrounding fat planes and mild-to-moderate pelvic free fluid with a hyperechoic material floating in the fluid. Also, there is a focal hyperechoic area in the wall of a small segment of the terminal ilium, with loss of gut signature in the same segment of the terminal ilium. Abdomen and Pelvis CT with IV CM done showed mild to moderate hemoperitoneum with extravasation at site of appendectomy (Figure 1).

**Figure 2:**
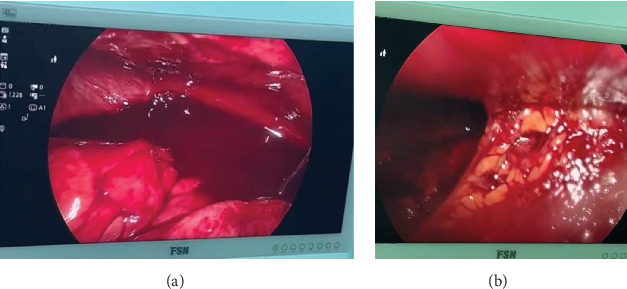
(A, B) Intraoperative images showed hemoperitoneum filling the peritoneum cavity and multiple adhesion bands at the right iliac fossa.

The patient was shifted to the operating room. Abdomen was opened via gridiron incision. The paracolic gutter was packed with purulent fluid. Mobilization of the cecum and the ascending colon disclosed an obscured area due to blood, not initially clear as to the appendiceal stump (Figure 2). The purulent fluid was drained, and the remaining appendiceal tissue was then identified and resected.

The postoperative recovery was uneventful, and the patient was discharged on the third day. The patient was stable and recovering well during the follow-up.

## 3. Discussion

Stump appendicitis is a rare complication where residual appendiceal tissue becomes inflamed after an initial appendectomy [4]. Though appendectomy is generally curative for appendicitis, stump appendicitis occurs due to incomplete removal of the appendix, leaving behind a remnant that can become obstructed and infected similarly to an intact appendix [6]. This condition poses a diagnostic challenge due to its low incidence and the assumption that appendectomy prevents recurrent appendicitis [7].

Often, stump appendicitis can mimic acute appendicitis; differential diagnoses for this condition are similar to those for appendicitis, such that we may suspect Crohn's ileitis, mesenteric adenitis, complicated cecal diverticulum, endometriosis, pelvic inflammatory disease, and gastroenteritis, in addition to any other differential diagnoses [8, 9]. These similarities can lead to delays in diagnosis and treatment, as recurrent appendicitis is not typically considered in postappendectomy patients presenting with abdominal pain.

Stump appendicitis is associated with late diagnosis, which makes us suspect that stump appendicitis is required in patients presenting with right lower quadrant abdominal pain and a surgical history of appendectomy [10]. Ultrasound or computed tomography abdominal imaging is diagnostic in most cases. Ultrasonography can show a thickened appendix stump, fluid in the right iliac fossa (RIF), and edema of the cecum

[11]. Computed tomography revealed dilated remnants of the appendiceal lumen, pericecal inflammatory infiltration, and abscess formation [12].

The standard treatment for stump appendicitis is completion of the appendectomy, even in the presence of perforation [2, 6]. In the situation where there is severe inflammation and the abscesses are involved, an ileocecectomy will be needed. Stump appendicitis can even be effectively managed laparoscopically, whether a perforation has occurred or not [13].

To our review, there are very few documented cases of stump appendicitis after 2 weeks or less of appendectomy. One such case, reported in 2013, described a 24-year-old man who was diagnosed with stump appendicitis after 24 h after surgery [14]. Another case reported in 2012 described a 33-year-old woman who was diagnosed with stump appendicitis after presented approximately 2 weeks following a laparoscopic appendectomy, complaining of RIF pain [15]. A further case reported in 2002 described a 24-year-old man diagnosed with stump appendicitis 4 days after a laparoscopic appendectomy [16].

In this case, a 17-year-old male presented with symptoms that could easily be mistaken for postsurgical complications rather than a separate infection of remaining appendiceal tissue. His initial diffuse abdominal pain that localized, high fever, and tenderness at the surgical site suggested an inflammatory or infectious process. However, the short time since his appendectomy (11 days) likely complicated the diagnostic process, as stump appendicitis typically develops months to years' postsurgery rather than days [3].

The ultrasound and CT findings further illustrate this complexity. Radiologic imaging highlighted abnormalities suggestive of infection and inflammation, such as thickened small bowel walls, increased echogenicity around the fat planes, free pelvic fluid, and hemoperitoneum. These findings, alongside the patient's elevated neutrophils and focal hyperechoic area in the terminal ileum, pointed toward residual inflammation, likely from the residual appendix stump. The CT also showed active extravasation at the surgical site, indicating hemorrhage, which might have masked the underlying stump and delayed its identification.

Upon exploratory surgery, the presence of purulent fluid and identification of residual appendiceal tissue confirmed stump appendicitis as the underlying cause. This case underscores the importance of thorough appendiceal resection during initial appendectomy, as well as the need for heightened awareness among clinicians regarding stump appendicitis, especially in patients with recurrent or persistent postappendectomy symptoms.

Early recognition and timely surgical intervention are critical in managing stump appendicitis, as delayed treatment can lead to complications like peritonitis or abscess formation [2, 8]. Fortunately, the patient had an uneventful recovery postsurgery, emphasizing the efficacy of prompt resection and drainage.

The limitation in this case was the unavailability of a histological image. However, the detailed report confirmed the presence of inflamed appendiceal tissue with transmural neutrophilic infiltration. This finding aligned with the patient's clinical symptoms, imaging results showing RLQ inflammation and hemoperitoneum, and intraoperative discovery of a purulent collection and retained inflamed stump. Together, these consistent findings confirmed the diagnosis.

## 4. Conclusion

Stump appendicitis is an uncommon but serious complication of appendectomy, most often presenting with vague symptoms, like right lower quadrant pain, nausea, and vomiting. Due to its uncommon nature, physicians have to consider stump appendicitis during the treatment course of a patient coming with abdominal pain and a history of appendectomy. Imaging—especially CT scans—at appropriate timing can help in making the correct diagnosis, while an appendectomy is the final line of treatment. It is a factor of prime priority that early detection and timely intervention hold much importance in order to keep away complications and get positive outcomes.

## Data Availability

The data that support the findings of this study are available from the corresponding author upon reasonable request.
